# Restrictions on Pesticides and Deliberate Self-Poisoning in Sri Lanka

**DOI:** 10.1001/jamanetworkopen.2024.26209

**Published:** 2024-08-06

**Authors:** Firouzeh Noghrehchi, Andrew H. Dawson, Jacques Raubenheimer, Fahim Mohamed, Indika B. Gawarammana, Michael Eddleston, Nicholas A. Buckley

**Affiliations:** 1 Translational Australian Clinical Toxicology, Faculty of Medicine and Health, The University of Sydney, Australia; 2 South Asian Clinical Toxicology Research Collaboration, Faculty of Medicine, University of Peradeniya, Sri Lanka; 3 Centre for Pesticide Suicide Prevention and Centre for Cardiovascular Science, University of Edinburgh, United Kingdom

## Abstract

**Importance:**

Deliberate self-poisoning using pesticides as a means of suicide is an important public health problem in low- and middle-income countries. Three highly toxic pesticides—dimethoate, fenthion, and paraquat—were removed from the market in Sri Lanka between 2008 and 2011. In 2015, less toxic pesticides (chlorpyrifos, glyphosate, carbofuran, and carbaryl) were restricted. Subsequent outcomes have not been well described.

**Objective:**

To explore the association of pesticide bans with pesticide self-poisonings and in-hospital deaths.

**Design, Setting, and Participants:**

In this cross-sectional study with an interrupted time series design, data were prospectively collected on all patients with deliberate self-poisonings presenting to 10 Sri Lankan hospitals between March 31, 2002, and December 31, 2019, and analyzed by aggregated types of poisoning. The correlates of pesticide bans were estimated within the pesticide group and on self-poisonings within other substance groups. The data analysis was performed between April 1, 2002, and December 31, 2019.

**Exposures:**

Implementation of 2 sets of pesticide bans.

**Main Outcomes and Measures:**

The main outcomes were changes in hospital presentations and in-hospital deaths related to pesticide self-poisoning as measured using segmented Poisson regression.

**Results:**

A total of 79 780 patients (median [IQR] age, 24 [18-34] years; 50.1% male) with self-poisoning from all causes were admitted to the study hospitals, with 29 389 poisonings (36.8%) due to pesticides. A total of 2859 patients died, 2084 (72.9%) of whom had ingested a pesticide. The first restrictions that targeted acutely toxic, highly hazardous pesticides were associated with an abrupt and sustained decline of the proportion of poisonings with pesticides (rate ratio [RR], 0.85; 95% CI, 0.78-0.92) over the study period and increases in poisonings with medications (RR, 1.11; 95% CI, 1.02-1.21) and household and industrial chemicals (RR, 1.20; 95% CI, 1.05-1.36). The overall case fatality of pesticides significantly decreased (RR, 0.33; 95% CI, 0.26-0.42) following the implementation of the 2008 to 2011 restrictions of highly hazardous pesticides. Following the 2015 restrictions of low-toxicity pesticides, hospitalizations were unchanged, and the number of deaths increased (RR, 1.98; 95% CI, 1.39-2.83).

**Conclusions and Relevance:**

These findings support the restriction of acutely toxic pesticides in resource-poor countries to help reduce hospitalization for and deaths from deliberate self-poisonings and caution against arbitrary bans of less toxic pesticides while more toxic pesticides remain available.

## Key Points

**Question:**

What is the association between bans of pesticides in Sri Lanka and deliberate self-poisoning hospitalizations and deaths?

**Findings:**

In this cross-sectional study of 79 780 patients hospitalized for deliberate self-poisoning, the percentage and case fatality of pesticide poisonings showed significant 18% and 67% declines, respectively, following bans of highly hazardous pesticides. Bans of low-toxicity pesticides were not associated with reductions in self-poisonings but were associated with increased case fatality.

**Meaning:**

These findings suggest that targeted pesticide bans in resource-poor countries may help to reduce hospitalizations for deliberate self-poisoning.

## Teaser

This cross-sectional study explores the association of pesticide bans with deliberate self-poisonings using pesticides and in-hospital deaths in Sri Lanka.

## Introduction

Suicide by deliberate self-poisoning using pesticides is a major but underrecognized public health problem in low- and middle-income countries (LMICs). Each year, between 110 000 and 168 000 people die from pesticide self-poisoning worldwide.^1^ These deaths are responsible for approximately 1 in 5 deaths by suicide globally^1^ and are particularly common in rural LMIC communities where acutely toxic, highly hazardous pesticides (HHPs) used for farming are readily available in people’s homes or from local shops.^2,3^ The World Health Organization recognizes pesticide poisoning to be 1 of the 3 most common means of suicide worldwide.^4^

An effective suicide prevention measure is to restrict access to means of lethal self-harm, such as pesticides, carbon monoxide, and firearms.^5,6,7,8^ Access to means is an important determinant to what is used in deliberate self-poisoning. Restriction of HHPs has been associated with a large reduction in deaths from pesticide self-poisoning in Sri Lanka^9^ and other countries.^6,10,11,12,13^ An association between pesticide restrictions and reductions in hospital admissions and in-hospital deaths has been shown in Sri Lanka^14,15^ and a few other countries.^16^

In Sri Lanka, a large prospective cohort study of hospital self-poisoning admissions showed that 75% of all fatal pesticide poisonings were due to only 3 pesticides: paraquat, dimethoate, and fenthion.^17^ A pilot study of the restriction of dimethoate and fenthion in a single provincial district showed a reduction in hospital admissions and a nonsustained reduction in overall pesticide case fatality.^15^ The demonstrated high toxicity of paraquat, dimethoate, and fenthion helped to inform a recommendation by the Pesticides Technical Advisory Committee to withdraw these 3 pesticides from Sri Lanka^18^ over a 3-year period, starting in 2008 for dimethoate and fenthion and 2009 for paraquat, by reducing the import quota by one-third each year. For reasons unrelated to acute toxicity, restrictions were subsequently implemented for 4 much less toxic pesticides, namely chlorpyrifos, glyphosate, carbofuran, and carbaryl, from 2013 to 2016, effective from 2015 (eTable 1 in Supplement 1).^19^ Previous literature has not reported an association of these bans with hospital outcomes. In addition, the outcomes of low-toxicity pesticide bans has not been investigated.

In this study, we examined the association of these pesticide bans with self-poisoning hospital admissions and in-hospital deaths within our long-term, prospective hospital-based cohort study. We also examined whether pesticide ingestion was replaced by ingestion of any other substances following these bans.

## Methods

This cross-sectional study received ethics approval from the research ethics committees of Colombo University Faculty of Medicine, Sri Lankan Medical Association, University of Peradeniya Faculty of Medicine, Australian National University, University of New South Wales, The University of Sydney, Oxfordshire Clinical Research Ethics Committee, and Oxford Tropical Medicine Ethics Committee. Patient informed consent was waived because this study used deidentified data. This study followed the Strengthening the Reporting of Observational Studies in Epidemiology (STROBE) reporting guideline.

### Patients

We examined data from the South Asian Clinical Toxicology Research Collaboration, a prospective observational cohort study of consecutive patients with deliberate ingestion of a pesticide who presented to 10 Sri Lankan study referral hospitals. A flowchart of patients and timeline of presentation are provided in eFigures 1 and 2 in Supplement 1. The study hospitals predominantly serve surrounding rural populations located in 4 agricultural provinces in Sri Lanka (central, northwestern, north central, and southern). There is no significant difference in demographics in these areas or the type of agriculture. Patients presenting to the study hospitals were very similar, being predominantly young (aged 18-34 years) rural residents who deliberately ingested pesticides. Patients were either directly admitted to the study hospital or transferred from smaller primary hospitals.

Patient recruitment commenced on March 31, 2002, with varying periods covered for the 10 hospitals since that time (eFigures 3 and 4; eTable 2 in Supplement 1). For our study, data analysis was performed for all patients who presented to a study hospital until December 31, 2019, including those patients reported in an earlier overall cohort analysis.^17^ Patients with primarily alcohol poisoning were excluded from this study because we considered alcohol unlikely to be chosen as an instrument of self-harm. Recruitment decreased substantially after 2016 due to decreased research funding, with fewer hospitals continuing recruitment thereafter.

### Procedures

Patients were enrolled into the cohort by clinical research assistants. Clinical care was determined by the treating physicians, patients were regularly reviewed, and major complications (eg, death) were recorded prospectively. The poison ingested was determined from the history given by the patient or relatives; the hospital transfer letter; the pesticide bottle, if available; or, occasionally, toxicologic analysis. The date of admission and data on patient age and sex were recorded on admission. More details on the procedures are presented elsewhere.^20,21,22^

### Statistical Analysis

We performed the data analysis between April 1, 2002, and December 31, 2019. Ingestions were aggregated and categorized as agricultural chemicals (pesticides), medicines, plants and fungi, household and industrial chemicals, or other or unknown ingestions. Pesticides were further categorized as herbicides; insecticides; or mixed, other, or unknown. Patients whose ingestion included substances from more than 1 category of pesticides were grouped with the mixed, other, or unknown category. Episodes in which patients ingested pesticides and/or any other nonpesticidal agents were identified. Cases in which multiple agents were taken were counted in each of their respective categories. Changes in poisoning epidemiology over time were examined by calculating the proportion of patients presenting within each substance category compared with all patients presenting. The outcomes were monthly proportion of presentations and examined in separate models for each substance category. This approach (ie, proportion of all poisonings) was chosen because (1) the sites were referral hospitals, and we did not have access to the number of the population at risk and (2) data collection periods varied across sites. The case fatality was defined as the proportion of deaths over admissions.

We used a quasi-experimental research design^23^ with interrupted time series analysis to evaluate the immediate impact and change in trends of poisonings before and after the 2 restrictions in January 2012 and January 2015. As restrictions for the first period were implemented incrementally after the announcement, we excluded the period between announcement and complete rollout (January 1, 2008, to December 31, 2011). The prerestriction period was April 1, 2002, to December 31, 2007. We excluded March 2002 admissions because the data collection started from March 31 and we aggregated data per month. There was no recruitment across the 10 study hospitals in April 2003 and May 2003, so these 2 months were removed from the analyses. The first postrestriction period was January 1, 2012, to December 31, 2019. The second postrestriction period was January 1, 2015, to December 31, 2019.

We analyzed the data using segmented Poisson regression or segmented negative binomial regression to account for overdispersion as necessary. Regression models included a time variable, an indicator variable for each postban status, and an interaction term between time and each indicator variable for postban status. The time variable indicates a gradual monthly trend in poisonings before the first restriction. The indicator for postban status estimates a change in level indicating an immediate and sustained change after restriction. The interaction between time and indicator variable for postban status estimates a change in slope indicating a gradual monthly change after restriction. Heterogeneity among hospitals was assessed using an intraclass correlation coefficient of 0.006. Autocorrelation was assessed and accounted for as necessary. To adjust for autocorrelation and heterogeneity, we used Newey-West and Driscoll-Kraay robust standard errors.^24,25^ To account for seasonality, we included Fourier terms. A 2-tailed *P* < .05 was considered to indicate statistical significance. The correlates of the 2 pesticide restrictions were examined in the pesticide category for (1) the total proportion of pesticide poisoning to all poisonings, (2) the proportion of poisonings due to each pesticide group, and (3) case fatality for pesticides. We also investigated the changes in the proportion of poisonings for each of the other substance groups. Results are presented as estimated rate ratios (RRs) and 95% CIs.

To assess the robustness of our analyses against the implemented robust standard errors, we conducted sensitivity analyses using segmented Poisson or negative binomial mixed-effects models with first-order autoregressive autocorrelation and controlled for clustering among hospitals. Because recruitment decreased after 2016, we conducted sensitivity analyses for the time between January 1, 2002, and December 31, 2016.

To investigate differences by age and sex, separate analyses for different sex and age groups were performed. All analyses were performed using R, version 4.0.1 (R Project for Statistical Computing). The eMethods in Supplement 1 provide additional details of the statistical analysis.

## Results

There were 79 780 patients in the study, 29 389 (36.8%) of whom ingested at least 1 of 234 generic pesticides. Of all patients, 49.9% were female and 50.1% male (eTable 3 in Supplement 1), and the median age was 24 years (IQR, 18-34 years). After initial assessment at small primary hospitals, 42.9% of all patients were transferred to the study hospital. Of patients who ingested at least 1 pesticide, 32.6% were female and 67.4% male, and the median age was 29 years (IQR, 21-41 years). We refer to patients, but it is probable that there are a few people who had more than 1 admission because the Sri Lankan hospital medical records are separate for each episode of care. More details on the cohort’s characteristics are presented elsewhere.^22^

Of all patients, 6029 (7.6%) ingested other or unknown substances, and 1597 (2.0%) ingested an unknown pesticide. There was a median of 143 (IQR, 114-178) pesticide poisonings per month prior to any restrictions. This number fell to 122.5 (IQR, 25-210) and 47.5 (IQR, 18-105) after the first and second ban, respectively (Table 1). The proportion of pesticide poisonings fell from 45.5% (95% CI, 44.8%-46.1%) to 27.5% (95% CI, 27.0%-27.9%) and 26.3% (95% CI, 25.6%-27.1%) after the first and second ban, respectively.

**Table 1.** Self-Poisonings by Substance Category

Category	Before ban (67 mo)^a^		Washout (48 mo)		First ban (96 mo)		Second ban (60 mo)		Total, No. (%)
	No. (%)	Median (IQR)	No. (%)	Median (IQR)	No. (%)	Median (IQR)	No. (%)	Median (IQR)	
All self-poisonings	21 606 (100)	278 (246-378)	23 476 (100)	410 (392-580)	42 771 (100)	374 (120-770)	14 165 (100)	148 (105-363)	87 853 (100)
Agricultural chemical	9834 (45.5)	143 (114-178)	8254 (35.2)	169 (137-198)	11 747 (27.5)	122.5 (25-210)	3730 (26.3)	47.5 (18-105)	29 835 (34.0)
Household and industrial chemicals	1139 (5.3)	13 (10-23)	1870 (8.0)	35 (30-46)	3273 (7.7)	20 (7-68)	736 (5.2)	9 (5-16)	6282 (7.1)
Medication	4875 (22.6)	49 (38-76)	9013 (38.4)	166.5 (144-227)	19 547 (45.7)	180 (80-328)	7089 (50.0)	91 (68-173)	33 435 (38.0)
Plant and fungus	4821 (22.3)	69 (59-81)	2496 (10.6)	46 (32-63)	4951 (11.6)	45 (9-90)	1449 (10.2)	17 (4-43)	12 268 (14.0)
Other or unknown	937 (4.3)	12 (7-21)	1843 (7.9)	38.5 (28-47)	3253 (7.6)	31 (11-51)	1161 (8.2)	15 (6-29)	6033 (6.9)
Proportional to all poisonings									
Herbicide	3021 (14.0)	44 (26-52)	2984 (12.7)	60 (51-72)	2916 (6.8)	23 (4-56)	710 (5.0)	5 (3-18)	8921 (10.1)
Insecticide	6353 (29.4)	95 (74-118)	4844 (20.6)	98 (72-119)	7989 (18.7)	78 (18-137)	2706 (19.1)	37 (12-74)	19 186 (21.8)
Mixed, other, or unknown	462 (2.1)	6 (6.5)	426 (1.8)	9 (7)	842 (2.0)	8 (10.5)	314 (2.2)	4 (6)	1730 (2.0)
Proportional to agricultural chemicals									
Herbicide	3021 (30.7)	As above	2984 (36.2)	As above	2916 (24.8)	As above	2706 (19.0)	As above	8921 (29.9)
Insecticide	6353 (64.6)	As above	4844 (58.7)	As above	7989 (68.0)	As above	2706 (72.5)	As above	19 186 (64.3)
Mixed, other, or unknown	462 (4.7)	As above	426 (5.2)	As above	842 (7.2)	As above	314 (8.4)	As above	1730 (5.8)

^a^ There was no data collection in April 2003 and May 2003; therefore, results are calculated over the remaining months. Numbers are estimated for prior to any pesticide ban and announcement (before January 1, 2012), during the washout period (between January 1, 2008, and December 31, 2011), after implementation of the first ban (between January 1, 2012, and December 31, 2019), and after implementation of the second ban (between January 1, 2015, and December 31, 2019).

A total of 2859 patient deaths were recorded, 2084 (72.9%) of which involved ingesting a pesticide. Deaths were recorded against 3.6% (217 of 6033) of the other or unknown substance poisonings and 2.2% (35 of 1597) of the unknown pesticide poisonings. The overall case fatality fell from 6.5% (95% CI, 6.2%-6.9%) prior to any restrictions to 1.7% (95% CI, 1.5%-1.9%) and 2.0% (95% CI, 1.7%-2.2%) after the first and second ban, respectively (eTable 4 in Supplement 1). The pesticide case fatality fell less dramatically from 10.9% (95% CI, 10.3%-11.5%) to 3.9% (95% CI, 3.6%-4.3%) and 4.9% (95% CI, 4.2%-5.6%). Pesticides responsible for most of the 1676 deaths in 2002-2012 were paraquat ( 585 deaths [34.9%]), dimethoate (200 deaths [11.9%]), and chlorpyrifos (175 deaths [10.4%]) in contrast to 2015 to 2019, when most of the 460 deaths were caused by unknown organophosphate (47 deaths [10.2%]) and profenofos (34 deaths [7.4%]).

**Figure 1.**
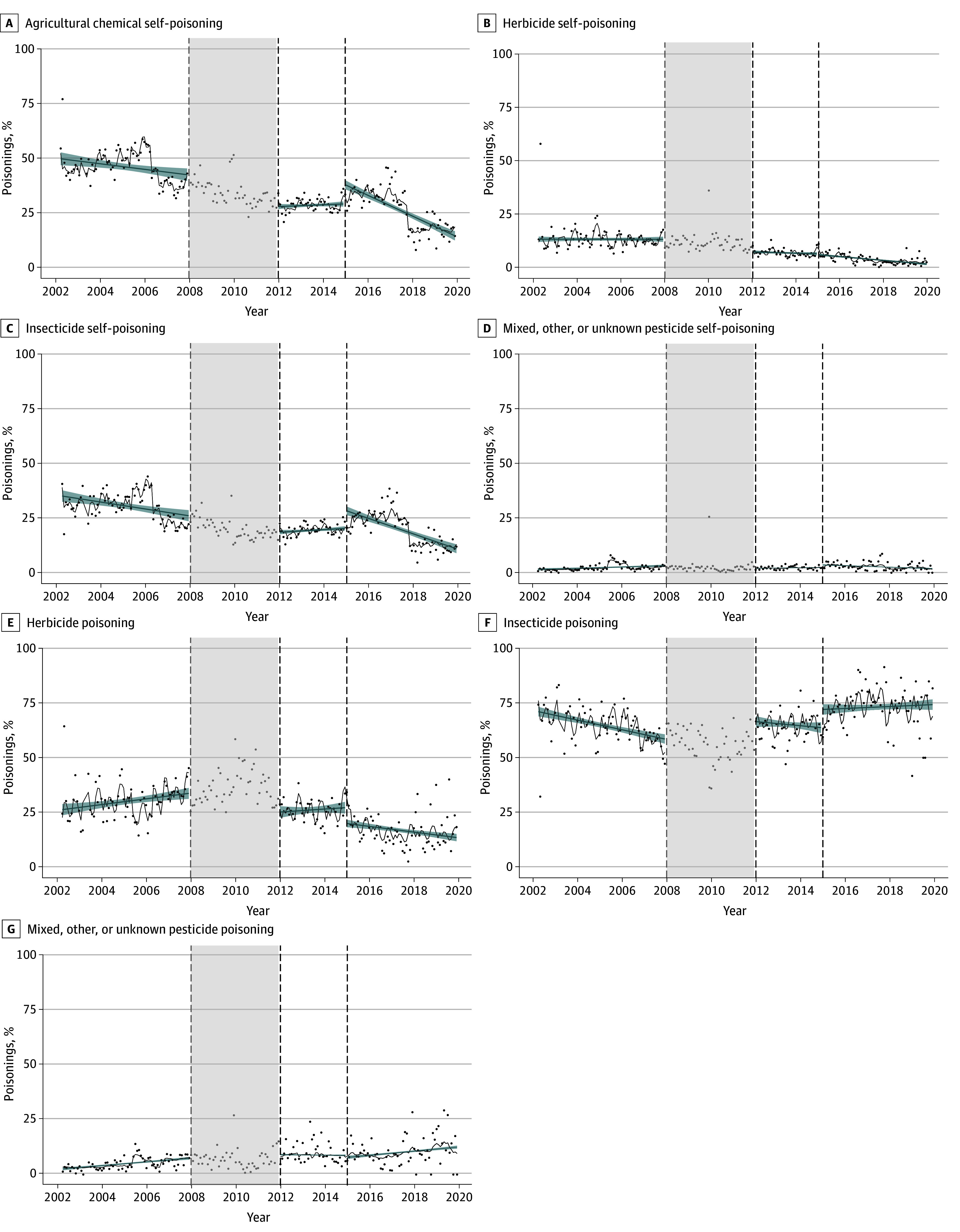
Monthly Proportion of Self-Poisonings With Pesticides Before and After the Implementation of Pesticide Bans in Sri Lanka (A) The proportion of pesticide poisoning to all self-poisonings. (B-D) The proportion of pesticide poisoning to all self-poisonings by pesticide subgroups. (E-G) The proportion of pesticide poisoning by pesticide subgroups. Points indicate mean monthly data across sites; dashed lines, time of restrictions; shaded areas between dashed lines, the start of first restrictions (washout period); black lines, expected values from the segmented Poisson regression model; and blue lines, linear smoothing splines of the expected values, with shading indicating associated 95% CIs.

The overall rate for pesticide self-poisoning significantly decreased over the 18-year study period, with a big step change at the time of first restrictions focused on acutely toxic HHPs (RR, 0.85; 95% CI, 0.78-0.92) (Table 2), but no change was observed when subsequent restrictions were done for reasons unrelated to self-poisoning. A shift to household and industrial chemical ingestion was seen after the first restrictions (from 5.3% to 7.7%; RR, 1.20; 95% CI, 1.05-1.36). Adjusted results for age and sex further showed a shift to medication ingestion (from 22.6% to 50.0%; RR, 1.11; 95% CI, 1.02-1.21) after the first restrictions (Table 1; Table 2; eFigure 5 in Supplement 1). Separate analyses by age and sex showed that these shifts mainly occurred in men younger than 25 years, with older women and younger men experiencing the greatest decreases in self-poisoning after the first restrictions (eTable 5 in Supplement 1). Proportional to all poisonings, there was a decrease in both herbicide (RR, 0.69; 95% CI, 0.61-0.78) and insecticide (RR, 0.89; 95% CI, 0.81-0.99) poisonings after the first restrictions (Table 2; Figure 1). Within pesticide groups, the proportion of self-poisoning from insecticide ingestions increased after the first restrictions (RR, 1.11; 95% CI, 1.07-1.15), while the proportion of self-poisoning from herbicide ingestion decreased (RR, 0.79; 95% CI, 0.71-0.87).

**Table 2.** Segmented Poisson Regression of Changes in the Monthly Number of Self-Poisonings After Implementation of the Pesticide Bans^a^

Variable	RR (95% CI)			
	Change after first restrictions		Change after second restrictions	
	Level	Slope	Level	Slope
Proportional to agricultural chemicals				
Herbicide	0.79 (0.71-0.87)^b^	1.00 (0.99-1.00)	0.99 (0.86-1.14)	0.99 (0.98-1.00)
Insecticide	1.11 (1.07-1.15)^b^	1.00 (1.00-1.00)	0.99 (0.93-1.05)	1.00 (1.00-1.01)
Mixed, other, or unknown^c^	1.05 (0.80-1.38)	1.00 (0.99-1.01)	1.14 (0.87-1.49)	1.00 (0.99-1.01)
Proportional to all poisonings				
Herbicide	0.69 (0.61-0.78)^b^	0.99 (0.99-1.00)	1.04 (0.87-1.23)	0.99 (0.99-1.01)
Insecticide	0.89 (0.81-0.99)^b^	1.00 (0.99-1.00)	0.99 (0.87-1.11)	1.00 (1.00-1.01)
Mixed, other, or unknown	0.80 (0.59-1.09)	0.99 (0.99-1.00)	1.15 (0.87-1.50)	1.00 (0.99-1.01)
Agricultural chemical	0.82 (0.75-0.90)^b^	1.00 (0.99-1.00)	1.01 (0.93-1.10)	1.00 (1.00-1.00)
Household and industrial chemicals	1.21 (1.06-1.38)^b^	1.00 (1.00-1.01)	0.95 (0.82-1.10)	1.00 (0.99-1.01)
Medication	1.10 (0.99-1.22)	0.99 (0.98-0.99)	1.05 (0.97-1.12)	1.00 (1.00-1.00)
Plant and fungus	0.90 (0.79-1.03)	1.01 (1.00-1.01)	1.03 (0.90-1.18)	1.00 (0.99-1.01)
Other or unknown	1.27 (0.95-1.70)	1.00 (0.99-1.02)	0.92 (0.69-1.23)	0.99 (0.97-1.00)
Adjusted model^d^				
Agricultural chemical	0.85 (0.78-0.92)^b^	0.99 (0.99-1.00)	1.02 (0.95-1.10)	1.00 (1.00-1.00)
Household and industrial chemicals	1.20 (1.05-1.36)^b^	1.00 (1.00-1.01)	0.95 (0.82-1.11)	0.99 (0.99-1.00)
Medication	1.11 (1.02-1.21)^b^	0.99 (0.99-1.00)	1.03 (0.96-1.10)	1.00 (1.00-1.00)
Plant and fungus	0.87 (0.76-1.00)	1.01 (1.00-1.01)	1.05 (0.92-1.19)	1.00 (0.99-1.01)
Other or unknown	1.32 (0.96-1.82)	1.00 (0.98-1.01)	0.94 (0.71-1.26)	0.99 (0.98-1.00)

Abbreviation: RR, rate ratio.

^a^ The outcome event was defined as the monthly proportion of self-poisoning presentations in the respective substance category. Outcome events were investigated in separate models, ie, 1 model per row.

^b^ Statistically significant.

^c^ Agricultural chemical ingestions were categorized as herbicides; insecticides; or mixed, other, or unknown. Patients who ingested substances from more than 1 category of agricultural chemicals were grouped with mixed, other, or unknown.

^d^ Adjusted for age and sex.

**Figure 2.**
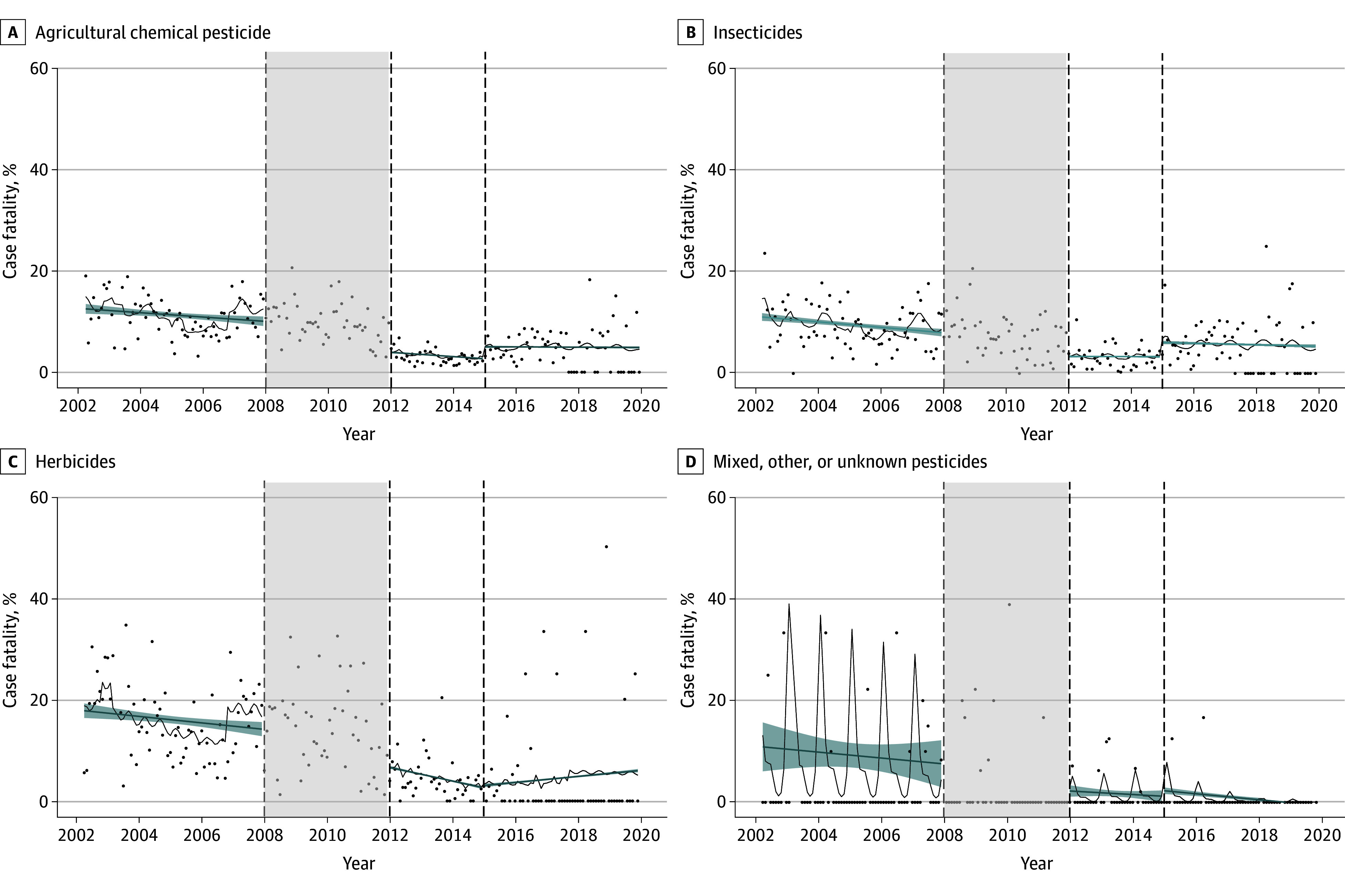
Monthly Case Fatality of Self-Poisonings With Pesticides Before and After the Implementation of Pesticide Bans in Sri Lanka Points indicate mean monthly data across sites; dashed lines, time of restrictions; shaded area between dashed lines, the start of first restrictions (washout period); black lines, expected values from the segmented Poisson regression model; and blue lines, linear smoothing splines of the expected values, with shading indicating associated 95% CIs.

The overall case fatality for pesticide self-poisoning significantly decreased over the 18-year study period, with a big step change at the time of first restrictions (RR, 0.33; 95% CI, 0.26-0.42) (Table 3; Figure 2). This decrease was due to a decrease in case fatality for both herbicides (RR, 0.39; 95% CI, 0.25-0.59) and insecticides (RR, 0.32; 95% CI, 0.24-0.43). The overall case fatality for pesticide poisoning increased at the time of the second restrictions (RR, 1.98; 95% CI, 1.39-2.83) compared with before the second restrictions; ie, it decreased at a lower rate (RR, 0.62; 95% CI, 0.45-0.86) compared with baseline. There was a monthly downward trend in case fatality for pesticides before the bans (RR, 0.99; 95% CI, 0.99-1.00), which was not altered by the restrictions of more toxic agents. The results from sensitivity analyses resembled the main results (eTables 6-10 in Supplement 1).

**Table 3.** Segmented Poisson Regression of Changes in the Monthly Case Fatality for Pesticides After the Pesticide Ban Implementation^a^

Variable	RR (95% CI)				
	Change after first restrictions		Change after second restrictions	
	Level	Slope	Level	Slope
Agricultural chemical	0.33 (0.26-0.42)^b^	1.00 (0.99-1.01)	1.98 (1.39-2.83)^b^	1.00 (0.99-1.01)
Herbicide	0.39 (0.25-0.59)^b^	0.99 (0.97-1.01)	1.27 (0.76-2.14)	1.02 (1.00-1.04)
Insecticide	0.32 (0.24-0.43)^b^	1.01 (0.99-1.02)	2.08 (1.36-3.18)^b^	0.99 (0.98-1.01)
Mixed, other, or unknown^c^	1.08 (0.22-5.27)	0.98 (0.92-1.05)	2.51 (0.38-16.00)^d^	0.97 (0.90-1.05)

Abbreviation: RR, rate ratio.

^a^ The outcome event was defined as the monthly case fatality of self-poisoning in the respective substance category. Outcome events were investigated in separate models, ie, 1 model per row.

^b^ Statistically significant.

^c^ Agricultural chemical ingestions were categorized as herbicides; insecticides; or mixed, other, or unknown. Patients who ingested substances from more than 1 category of agricultural chemicals were grouped with mixed, other, or unknown.

^d^ Large confidence interval due to a low number of deaths in this category.

## Discussion

This cross-sectional study is based on one of the most detailed long-term prospective cohort studies to investigate the association of pesticide bans with hospital admissions for self-poisoning and in-hospital deaths. We analyzed systematically collected data of 79 780 patients presenting to 10 rural hospitals, with a mix of 234 generic pesticides ingested by 29 389 patients. The findings of our study suggest that declines in the hospital presentation of pesticide self-poisonings in Sri Lanka^9^ may be attributable to effective implementation of bans of acutely toxic HHPs. The results also show that targeted restrictions were associated with reduced case fatality for pesticides. The reductions in poisoning and case fatality with herbicides may be due to the paraquat ban, and reductions in poisoning with insecticides may be due to bans of organophosphates fenthion and dimethoate. The improved case fatality for pesticides over time in Sri Lanka suggest that medical management may have improved within our cohort as well. We also provide evidence that arbitrary bans of less toxic pesticides are not associated with reduced self-poisoning, possibly because people ingesting low-toxicity pesticides may have been less likely to have severe poisoning and be admitted to the hospital to begin with. Furthermore, low-toxicity pesticide bans were not followed by a switch to nonpesticide poisonings and may increase the number of deaths, suggesting a shift to more toxic pesticides. Therefore, accurate targeting of the most hazardous available pesticides is required for restrictions to be effective in reducing deaths by suicide attributable to poisoning. When considering restrictions of low-toxicity pesticides, regulators should consider the acute toxicity of the agricultural replacements in countries where pesticide self-poisoning is a substantial problem.

The long-term success of targeted pesticide bans in reducing overall in-hospital case fatality of poisoning is also seen in our data. There has also been a 70% reduction in suicide and an even greater decline in fatal self-poisoning following the targeted pesticide bans.^9^ We speculate that further targeted bans of pesticides may lead to further modest reductions in fatal poisonings and suicides. Carbosulfan, in particular, is responsible for 60% of all pesticide deaths.^26^ Profenofos also very commonly causes prolonged respiratory failure, resulting in high morbidity.^27^ Several other Asian countries have had remarkable reductions in fatal pesticide poisoning coinciding with bans of more toxic agents.^6,28^ There may be many rural LMICs that are less well resourced, where HHPs may be associated with even higher case fatality and the gains may be even greater. Targeted pesticide restrictions do not have apparent adverse effects on agricultural costs or output.^6^

Over time and partly associated with pesticide bans, there has been a large increase in the proportion of medication poisonings (from 22.6% to 50.0%), which are much less likely to be fatal. The first but not the second restriction was followed by a shift in agents away from poisoning by pesticides and toward medication and other agents. This shift contributed to the very large overall reduction in case fatality for all poisonings. These findings may be coincidental, as many factors may have increased medication use and availability between 2008 and 2012. However, they may also reflect greater community and vendor awareness of self-poisoning risks and resulting precautions coinciding with the bans.

### Limitations

Our study has several limitations. First, the study hospitals were secondary referral hospitals; deaths at home, in primary hospitals, and during transfers would not have been recorded. Most deaths from pesticide poisoning occur in the secondary referral hospital^29^ and not rapidly, with typical times to in-hospital death of more than 24 hours.^27,30,31^ Patients who were well and not transferred would have reduced the denominators; however, in Sri Lanka, approximately 80% to 90% of patients with pesticide poisoning presenting to small hospitals are rapidly transferred.^32^ Thus, this issue would largely apply for agents recognized to have very low toxicity and would have only a very small effect on case-fatality estimates for pesticides and other more toxic agents.^29^

Second, the number of hospitals recruiting to the cohort increased or decreased over time depending on funding, and this may have influenced the types of ingested agents observed. Although data on all poisonings were captured, there is a potential for selection bias. We have applied robust standard errors and sensitivity analyses with mixed-effects models to reduce this potential bias.^33^

Third, the restriction of HHPs preceded the restriction of low-toxicity pesticides. It is not clear whether and how the outcomes of the first ban are associated with the second. If anything, the first ban may have led to an increase in chlorpyrifos use. If the less toxic pesticides were banned while the more hazardous pesticides were still available, the outcomes may have been much worse.

Finally, because the bans were a national legislative change, we had no control population. Interrupted time series analysis is a powerful quasi-experimental method that can identify a causal effect if there are no other changes at the time of intervention that could affect the outcome. To our knowledge, there were no other changes around the time of the pesticide bans that may have contributed to poisoning-related hospitalizations in the 10 study sites. Thus, our analysis of changes in self-poisonings with other substances suggests that the targeted pesticide bans in Sri Lanka may have reduced hospitalizations for pesticide poisoning. Nonetheless, analysis of an unrelated outcome could add a negative control to investigate the causal effect of this policy change. Future studies could investigate the why and how of the association and whether there are differences in outcomes, eg, shifts to other substance categories, based on social determinants of health other than patient age and sex.

## Conclusions

The findings from this cross-sectional study support moves to completely eliminate pesticides that have a case fatality of more than 5%, as obtained from human data on acute toxicity,^22^ and caution against restriction of less toxic pesticides while more toxic pesticides remain available. Coordinated regulation of all pesticides with high case fatality might lead to a large and rapid reduction of deaths from pesticide poisoning worldwide.

## Notes

Supplement 1.
eMethods

eReferences
**eTable 1.** Dates of Initiation of Phased-In Partial Restrictions and Subsequent Importation Bans and Legislative Restrictions on Pesticides in Sri Lanka **eTable 2.** Number of Patients by Study Hospitals Over Time **eTable 3.** Characteristics of Cohort, Overall and by Time Period **eTable 4.** Case Fatality (95% CI) of Different Pesticides Over Time **eTable 5.** Segmented Poisson Regression to Investigate Changes in the Monthly Number of Self-Poisonings After the Implementation of Pesticide Bans, Stratified by Age and Sex **eTable 6.** Segmented Poisson Mixed-Effects Regression With AR(1) to Investigate Changes in the Monthly Number of Self-Poisonings After the Implementation of Pesticide Bans **eTable 7.** Adjusted Segmented Poisson Mixed-Effects Regression With AR(1) to Investigate Changes in the Monthly Number of Self-Poisonings After the Implementation of Pesticide Bans, Adjusted for Age and Sex **eTable 8.** Segmented Poisson Mixed-Effects Regression With AR(1) to Investigate Changes in the Monthly Case Fatality of Pesticide Groups After the Implementation of Bans **eTable 9.** Segmented Poisson Regression to Investigate Changes in the Monthly Number of Self-Poisonings After the Implementation of Pesticide Bans, Between January 2002 and December 2016 **eTable 10.** Segmented Poisson regression to Investigate Changes in the Monthly Case-Fatality of Pesticide Groups After the Implementation of Bans, Between January 2002 and December 2016 **eFigure 1.** Flowchart of Patients in the Study **eFigure 2.** Timeline of Patient Recruitment Over Time **eFigure 3.** Patient Recruitment Commenced on March 31, 2002, With Varying Periods Covered for the 10 Hospitals **eFigure 4.** Self-Poisoning Over Time by Different Hospital Sites **eFigure 5.** Monthly Proportion of Self-Poisonings With Nonpesticides Before and After the Implementation of Pesticide Bans in Sri Lanka

Supplement 2.
Data Sharing Statement
