# Supplementary material for: Restrictions on Pesticides and Deliberate Self-Poisoning in Sri Lanka
Source: JAMA Netw Open. 2024 Aug 6;7(8):e2426209. doi: 10.1001/jamanetworkopen.2024.26209 (PMC11304112; doi:10.1001/jamanetworkopen.2024.26209)
Supplement: Supplement 1. — eMethods eReferences eTable 1. Dates of Initiation of Phased-In Partial Restrictions and Subsequent Importation Bans and Legislative Restrictions on Pesticides in Sri Lanka eTable 2. Number of Patients by Study Hospitals Over Time eTable 3. Characteristics of Cohort, Overall and by Time Period eTable 4. Case Fatality (95% CI) of Different Pesticides Over Time eTable 5. Segmented Poisson Regression to Investigate Changes in the Monthly Number of Self-Poisonings After the Implementation of Pesticide Bans, Stratified by Age and Sex eTable 6. Segmented Poisson Mixed-Effects Regression With AR(1) to Investigate Changes in the Monthly Number of Self-Poisonings After the Implementation of Pesticide Bans eTable 7. Adjusted Segmented Poisson Mixed-Effects Regression With AR(1) to Investigate Changes in the Monthly Number of Self-Poisonings After the Implementation of Pesticide Bans, Adjusted for Age and Sex eTable 8. Segmented Poisson Mixed-Effects Regression With AR(1) to Investigate Changes in the Monthly Case Fatality of Pesticide Groups After the Implementation of Bans eTable 9. Segmented Poisson Regression to Investigate Changes in the Monthly Number of Self-Poisonings After the Implementation of Pesticide Bans, Between January 2002 and December 2016 eTable 10. Segmented Poisson regression to Investigate Changes in the Monthly Case-Fatality of Pesticide Groups After the Implementation of Bans, Between January 2002 and December 2016 eFigure 1. Flowchart of Patients in the Study eFigure 2. Timeline of Patient Recruitment Over Time eFigure 3. Patient Recruitment Commenced on March 31, 2002, With Varying Periods Covered for the 10 Hospitals eFigure 4. Self-Poisoning Over Time by Different Hospital Sites eFigure 5. Monthly Proportion of Self-Poisonings With Nonpesticides Before and After the Implementation of Pesticide Bans in Sri Lanka [file jamanetwopen-e2426209-s001.pdf]

Supplemental Online Content

Noghrehchi F, Dawson AH, Raubenheimer J, et al. Restrictions on pesticides and deliberate self-poisoning in Sri Lanka. *JAMA Netw Open*. 2024;7(8):e2426209. doi:10.1001/jamanetworkopen.2024.26209

eMethods.

eReferences.

eTable 1. Dates of Initiation of Phased-In Partial Restrictions and Subsequent Importation Bans and Legislative Restrictions on Pesticides in Sri Lanka

eTable 2. Number of Patients by Study Hospitals Over Time

eTable 3. Characteristics of Cohort, Overall and by Time Period

eTable 4. Case Fatality (95% CI) of Different Pesticides Over Time

eTable 5. Segmented Poisson Regression to Investigate Changes in the Monthly Number of Self-Poisonings After the Implementation of Pesticide Bans, Stratified by Age and Sex

eTable 6. Segmented Poisson Mixed-Effects Regression With AR(1) to Investigate Changes in the Monthly Number of Self-Poisonings After the Implementation of Pesticide Bans

eTable 7. Adjusted Segmented Poisson Mixed-Effects Regression With AR(1) to Investigate Changes in the Monthly Number of Self-Poisonings After the Implementation of Pesticide Bans, Adjusted for Age and Sex

eTable 8. Segmented Poisson Mixed-Effects Regression With AR(1) to Investigate Changes in the Monthly Case Fatality of Pesticide Groups After the Implementation of Bans

eTable 9. Segmented Poisson Regression to Investigate Changes in the Monthly Number of Self-Poisonings After the Implementation of Pesticide Bans, Between January 2002 and December 2016

eTable 10. Segmented Poisson regression to Investigate Changes in the Monthly Case-Fatality of Pesticide Groups After the Implementation of Bans, Between January 2002 and December 2016

eFigure 1. Flowchart of Patients in the Study

eFigure 2. Timeline of Patient Recruitment Over Time

eFigure 3. Patient Recruitment Commenced on March 31, 2002, With Varying Periods Covered for the 10 Hospitals

eFigure 4. Self-Poisoning Over Time by Different Hospital Sites

eFigure 5. Monthly Proportion of Self-Poisonings With Nonpesticides Before and After the Implementation of Pesticide Bans in Sri Lanka

This supplemental material has been provided by the authors to give readers additional information about their work.

© 2024 Noghrehchi F et al. *JAMA Network Open.*

eMethods

1. Interrupted time series analysis

We performed an interrupted time series analysis to quantify changes in monthly poisonings after two pesticide bans in January 2012 and January 2015, respectively. See Supplementary Table 1 for detailed timeline of effective restrictions.

Interrupted time series analysis is one of the strongest observational study designs for evaluating the impact of population-level interventions.

Our data are counts (i.e., number of poisoning admissions), with λ denoting the expected counts. Thus, we modelled the intervention using a segmented Poisson or negative binomial regression.

The base segmented regression model can be expressed as:

$$\log(\lambda_t) = \beta_0 + \beta_1 \times time + \beta_2 \times 1^{st}\ intervention + \beta_3 \times time\ since\ 1^{st}\ intervention + \beta_4 \times 2^{nd}\ intervention + \beta_5 \times time\ since\ 2^{nd}\ intervention,$$

where

- λ_t is the expected number of poisoning-related hospital admissions at time *t*.
- β_0 is the intercept, or Y_t at time zero.
- β_1 is the slope (monthly trend) pre-interventions or change in the number of poisonings per month.
- *time* is an integer representing the number of months from the start of the study.
- β_2 and β_4 represent the level change post 1st intervention in January 2012 and 2nd intervention in January 2015, respectively. These coefficients give immediate and sustained change after intervention for the duration of the study period i.e., difference between observed and predicted values based on pre-intervention trend.
- $intervention$ is a dichotomous variable that takes the value of "0" prior to intervention and "1" otherwise.

© 2024 Noghrehchi F et al. *JAMA Network Open.*

- β_3 and β_5 are the changes in slope, indicating a gradual monthly change in poisonings after 1st ban and 2nd ban, respectively.[1,2]

- $time\ since\ intervention$ is an integer taking the value of "0" prior to intervention and increasing by 1 from the date of intervention.

A regression model assumes that the errors are independent i.e., not serially correlated. However, this assumption can potentially be violated in time series data when residual autocorrelation and/or seasonality is present. The interrupted time series models included hospital fixed effects. Inferences were computed with robust standard errors to account for heteroscedasticity and autocorrelation. We used a combination of the Durbin-Watson test, and the autocorrelation function and partial autocorrelation function plots to test for the presence of autocorrelation within hospital sites in our models. If any of these tests indicated that autocorrelation is present, we used Newey-West and Driscoll and Kraay standard errors, using the *vcovPL* function from the *sandwich* R-package, to adjust for autocorrelation within clusters (hospital sites).[3] Autocorrelation was detected in all statistical tests.

We investigated for seasonality by (1) including no seasonality, (2) including dummy variables representing the months and (3) including Fourier terms in our models. The dummy variable for each month takes a value of "1" in that month and "0" otherwise. The Fourier terms were included in the form of $\sin(2\pi t/12)$ and $\cos(2\pi t/12)$ pairs to account for monthly data. We used the Akaike Information Criterion (AIC) to choose the most appropriate model for seasonality. The model with the lowest AIC was deemed as the most parsimonious suitable model. Based on AIC, seasonality in the form of Fourier terms was added to the models for all outcomes.

To account for overdispersion, we used combination of checking residual deviance by dividing the residual deviance by the degrees of freedom, and hypothesis testing by using the *dispersiontest* function from the *AER* R-package.[4,5] If overdispersion was detected in a model based on statistical test and large residual deviance ratio, we applied negative binomial regression. All the analyses were performed in R version 4.0.1.

We further investigated the changes in self-poisoning after pesticide bans on the number of poisonings with pesticide and other substance groups after adjusting for age and sex. Age was defined in 3 categories of 0–24, 25–64 years and ≥ 65 years. We also investigated these changes stratified by sex and age (Supplementary Table 5).

© 2024 Noghrehchi F et al. *JAMA Network Open.*

2. Sensitivity analysis

For sensitivity, we investigated the changes in self-poisoning (Supplementary Table 6 and 7) and case-fatality (Supplementary Table 8) using segmented mixed-effect models with autoregressive of order 1 and accounting for clustering effect of hospital sites. Mixed-effect models were performed using the *glmmTMB* function from the *glmmTMB* R-package.[6]

To assess the robustness of our analyses against any potential bias related to the recruitment decrease after 2016, we did further sensitivity analyses where December 2016 was defined as the end date for the study, totaling 60 months and 24 months post-intervention after the first bans and the second bans, respectively. Using segmented Poisson regression, we investigated the changes in self-poisoning (Supplementary Table 9) and case-fatality (Supplementary Table 10) for the time between January 2002 and December 2016.

© 2024 Noghrehchi F et al. *JAMA Network Open.*

eReferences

1. Wagner AK, Soumerai SB, Zhang F, Ross-Degnan D. Segmented regression analysis of interrupted time series studies in medication use research. *Journal of clinical pharmacy and therapeutics*. 2002. Aug;27(4):299-309.

2. Penfold RB, Zhang F. Use of interrupted time series analysis in evaluating health care quality improvements. *Academic pediatrics*. 2013. Nov 1;13(6):S38-44.

3. Zeileis A, Köll S, Graham N. Various versatile variances: an object-oriented implementation of clustered covariances in R. *Journal of Statistical Software*. 2020. Oct 7;95:1-36.

4. Cameron AC, Trivedi PK. Microeconometrics: methods and applications. Cambridge university press; 2005 May 9.

5. Cameron AC, Trivedi PK. Regression analysis of count data. Cambridge university press; 2013 May 27.

6. Magnusson A, Skaug H, Nielsen A, Berg C, Kristensen K, Maechler M, van Bentham K, Bolker B, Brooks M, Brooks MM. Package 'glmmtmb'. R Package Version 0.2. 0. 2017. Dec 8;25.

7. Buckley NA, Fahim M, Raubenheimer J, Gawarammana IB, Eddleston M, Roberts MS, Dawson AH. Case fatality of agricultural pesticides after self-poisoning in Sri Lanka: a prospective cohort study. *Lancet Global Health*. 2021. Jun 1;9(6): e854-62.

8. Department of Agriculture. Performance Report 2015. Dept of Agriculture, Peradeniya, Sri Lanka; 2016.

9. Office of the Registrar of Pesticides. List of banned and severely restricted pesticides in Sri Lanka with the year of implementation and the year of legal declaration. 2020.

© 2024 Noghrehchi F et al. *JAMA Network Open.*

eTable 1. Dates of Initiation of Phased-In Partial Restrictions and Subsequent Importation Bans and Legislative Restrictions on Pesticides in Sri Lanka

Pesticide	Partial restriction/ Phase out period starts	Effective Restrictions	Legislative ban
**Most POPs**		1970-95	2001
**Class I pesticides**		1984-95	2001
**Endosulfan**		1998	2001
**Paraquat**	2008	2012	2014
**Dimethoate**	2008	2011	2014
**Fenthion**	2008	2011	2014
**Cyromazine**	?	2011	2014
**Alachlor**	?	2012	2014
**Glyphosate**	2014[a]	2015	2015[b]
**Carbofuran**	2013	2015	2016
**Carbaryl**	2013	2015	2016
**Chlorpyrifos**	2013	2015	2016
**Propanil**	2014[a]		-

[a] Regional restriction
[b] In 2018 reregistered with tight restrictions
Note: This table is acquired from our cohort description article.[7] Bolded agents restricted to reduce risks from acute poisoning, other restrictions for environmental or chronic toxicity.
Internet sources[8,9]: https://doa.gov.lk/SCPPC/images/ROP/Tabel.pdf
https://www.parliament.lk/uploads/documents/paperspresented/performance-report-department-of-agriculture-2015.pdf

© 2024 Noghrehchi F et al. *JAMA Network Open.*

eTable 2. Number of Patients by Study Hospitals Over Time

	Pre-ban		Washout		1st ban		2nd ban	
	No. of months	N (%)	No. of months	N (%)	No. of months	N (%)	No. of months	N (%)
**All patients**	67	19,867 (100)	48	21,437 (100)	96	25,941 (100)	60	12,535 (100)
**Anuradhapura**	65	9,809 (49.4)	48	8,351 (39.0)	54	6,105 (23.5)	18	2,323 (18.5)
**Chilaw**	16	889 (4.5)	27	1,072 (5.0)	34	955 (3.7)	0	0 (0)
**Galle**	14	1,275 (6.4)	48	4,958 (23.1)	38	3,374 (13.0)	2	64 (0.5)
**Kurunegala**	4	533 (2.7)	15	2,356 (11.0)	70	5,306 (20.5)	34	3,341 (26.7)
**Marawila**	0	0 (0)	4	75 (0.3)	34	802 (3.1)	0	0 (0)
**Matara**	0	0 (0)	17	1,160 (5.4)	39	2,715 (10.5)	3	150 (1.2)
**Nuwara Eliya**	11	486 (2.4)	0	0 (0)	52	491 (1.9)	34	1,129 (9.0)
**Peradeniya**	14	682 (3.4)	48	2,355 (11.0)	96	3,882 (15.0)	60	5,451 (43.5)
**Polonnaruwa**	66	6,193 (31.2)	19	1,005 (4.7)	38	1,701 (6.6)	3	77 (0.6)
**Puttalam**	0	0 (0)	4	105 (0.5)	34	610 (2.4)	0	0 (0)

© 2024 Noghrehchi F et al. *JAMA Network Open.*

eTable 3. Characteristics of Cohort, Overall and by Time Period

	**Pre-ban** (N=19867)	**Washout** (N=21437)	**1st ban** (N=25941)	**2nd ban** (N=12535)	**Overall** (N=79780)
**Sex**					
Female	9292 (46.8%)	11015 (51.4%)	13051 (50.3%)	6424 (51.2%)	39782 (49.9%)
Male	10575 (53.2%)	10420 (48.6%)	12890 (49.7%)	6111 (48.8%)	39996 (50.1%)
Unknown	0 (0%)	2 (0.0%)	0 (0%)	0 (0%)	2 (0.0%)
**Age (years)**					
Median (Q1, Q3)	24.0 (19.0, 35.0)	23.0 (18.3, 33.0)	23.0 (18.0, 33.0)	24.0 (19.0, 36.0)	24.0 (18.0, 34.0)
**Age group**					
0–24	10139 (51.0%)	11567 (54.0%)	14572 (56.2%)	6417 (51.2%)	42695 (53.5%)
25–64	9345 (47.0%)	9428 (44.0%)	10752 (41.4%)	5735 (45.8%)	35260 (44.2%)
≥ 65	382 (1.9%)	439 (2.0%)	616 (2.4%)	382 (3.0%)	1819 (2.3%)
Unknown	1 (0.0%)	3 (0.0%)	1 (0.0%)	1 (0.0%)	6 (0.0%)
**Time to admission (minutes)**					
Median (Q1, Q3)	--	195 (111, 360)	185 (100, 360)	210 (120, 395)	195 (110, 365)
Unknown	19867 (100%)	13068 (61.0%)	2247 (8.7%)	737 (5.9%)	35919 (45.0%)
**Transferred from primary hospital**					
Transferred	3079 (15.5%)	11682 (54.5%)	13284 (51.2%)	7674 (61.2%)	35719 (44.8%)

© 2024 Noghrehchi F et al. *JAMA Network Open.*

eTable 4. Case Fatality (95% CI) of Different Pesticides Over Time

	**Pre-ban** (67 months) Deaths = 1,069	**Washout** (48 months) Deaths = 607	**1st ban** (96 months) Deaths = 460	**2nd ban** (60 months) Deaths = 185
**All poisonings**	6.5 (6.2, 6.9)	3.5 (3.3, 3.8)	1.7 (1.5, 1.9)	2.0 (1.7, 2.2)
**Agricultural chemical**	10.9 (10.3, 11.5)	7.4 (6.8, 7.9)	3.9 (3.6, 4.3)	4.9 (4.2, 5.6)
**Herbicide**	14.9 (13.7, 16.3)	10.0 (8.9, 11.1)	4.2 (3.5, 5.0)	3.5 (2.3, 5.1)
**Insecticide**	9.3 (8.6, 10.1)	6.0 (5.4, 6.7)	4.1 (3.6, 4.5)	5.7 (4.9, 6.7)
**Mixed/other/unknown**	5.6 (3.7, 8.1)	4.2 (2.6, 6.6)	1.4 (0.7, 2.5)	0.9 (0.2, 2.7)

© 2024 Noghrehchi F et al. *JAMA Network Open.*

eTable 5. Segmented Poisson Regression to Investigate Changes in the Monthly Number of Self-Poisonings After the Implementation of Pesticide Bans, Stratified by Age and Sex

	**Change after 1st restrictions in level** RR (95% CI)	**Change after 1st restrictions in slope** RR (95% CI)	**Change after 2nd restrictions in level** RR (95% CI)	**Change after 2nd restrictions in slope** RR (95% CI)
**Female, < 25 years**				
Agricultural chemical	**0.85 (0.72, 0.99)**	1.00 (0.99, 1.00)	0.96 (0.83, 1.11)	1.00 (0.99, 1.01)
Household & industrial chemical	1.15 (0.92, 1.45)	1.00 (0.99, 1.01)	1.07 (0.86, 1.33)	1.00 (0.99, 1.01)
Medication	1.02 (0.93, 1.12)	0.99 (0.99, 1.00)	1.02 (0.96, 1.09)	1.00 (1.00, 1.00)
Plant & fungus	0.79 (0.65, 1.00)	1.01 (1.00, 1.01)	1.06 (0.87, 1.30)	0.99 (0.98, 1.00)
Other/unknown	1.23 (0.80, 1.89)	0.99 (0.97, 1.01)	1.04 (0.67, 1.61)	0.99 (0.97, 1.01)
**Female, 25-64 years**				
Agricultural chemical	0.92 (0.78, 1.10)	0.99 (0.99, 1.00)	1.00 (0.86, 1.16)	1.00 (1.00, 1.01)
Household & industrial chemical	**1.34 (1.01, 1.77)**	1.01 (1.00, 1.02)	0.66 (0.49, 1.07)	1.00 (0.99, 1.02)
Medication	1.03 (0.91, 1.17)	1.00 (0.99, 1.00)	1.09 (1.00, 1.18)	1.00 (0.99, 1.00)
Plant & fungus	0.84 (0.69, 1.01)	1.00 (0.99, 1.01)	1.01 (0.78, 1.32)	1.00 (0.99, 1.01)
Other/unknown	1.07 (0.63, 1.83)	0.99 (0.96, 1.01)	0.89 (0.55, 1.44)	1.00 (0.97, 1.02)
**Female, ≥ 65 years**				
Agricultural chemical	**0.44 (0.21, 0.92)**	1.01 (0.98, 1.03)	0.92 (0.51, 1.66)	0.98 (0.95, 1.01)
Household & industrial chemical	0.88 (0.21, 3.69)	1.01 (0.96, 1.06)	1.68 (0.54, 5.21)	0.98 (0.93, 1.03)
Medication	1.57 (0.85, 2.90)	1.00 (0.98, 1.02)	0.86 (0.52, 1.43)	1.01 (1.00, 1.02)
Plant & fungus	2.22 (0.67, 7.34)	1.02 (0.98, 1.06)	1.12 (0.24, 5.17)	1.00 (0.95, 1.06)
Other/unknown	0.98 (0.46, 2.10)	0.97 (0.93, 1.01)	1.75 (0.48, 6.36)	1.00 (0.96, 1.04)
**Male, < 25 years**				
Agricultural chemical	**0.64 (0.55, 0.74)**	0.99 (0.99, 1.00)	1.04 (0.90, 1.21)	1.00 (0.99, 1.00)

© 2024 Noghrehchi F et al. *JAMA Network Open.*

	Change after 1st restrictions in level RR (95% CI)	Change after 1st restrictions in slope RR (95% CI)	Change after 2nd restrictions in level RR (95% CI)	Change after 2nd restrictions in slope RR (95% CI)
Household & industrial chemical	**1.71 (1.38, 2.13)**	0.99 (0.98, 1.00)	1.24 (0.94, 1.62)	0.99 (0.97, 1.00)
Medication	**1.30 (1.07, 1.57)**	0.98 (0.97, 1.00)	1.01 (0.90, 1.13)	1.00 (1.00, 1.01)
Plant & fungus	0.91 (0.73, 1.12)	1.00 (1.00, 1.01)	1.13 (0.99, 1.30)	0.99 (0.98, 1.00)
Other/unknown	1.31 (0.95, 1.79)	1.01 (0.99, 1.02)	0.88 (0.67, 1.15)	0.98 (0.97, 1.00)
**Male, 25-64 years**				
Agricultural chemical	0.96 (0.89, 1.03)	0.99 (0.99, 1.00)	1.05 (0.97, 1.14)	1.00 (1.00, 1.01)
Household & industrial chemical	0.86 (0.68, 1.09)	1.01 (1.00, 1.02)	0.80 (0.53, 1.20)	0.99 (0.98, 1.00)
Medication	1.06 (0.89, 1.27)	1.00 (0.99, 1.01)	0.94 (0.8, 1.12)	1.00 (0.99, 1.00)
Plant & fungus	0.90 (0.75, 1.08)	1.01 (1.01, 1.02)	1.08 (0.87, 1.33)	1.00 (0.99, 1.01)
Other/unknown	**1.78 (1.28, 2.47)**	1.00 (0.98, 1.01)	1.04 (0.77, 1.40)	1.00 (0.98, 1.01)
**Male, ≥ 65 years**				
Agricultural chemical	0.98 (0.78, 1.22)	1.00 (0.98, 1.01)	1.05 (0.75, 1.46)	1.00 (0.99, 1.02)
Household & industrial chemical	0.64 (0.27, 1.52)	0.98 (0.95, 1.02)	0.65 (0.27, 1.55)	0.98 (0.94, 1.02)
Medication	1.37 (0.76, 2.44)	1.00 (0.97, 1.03)	1.07 (0.61, 1.87)	1.00 (0.98, 1.03)
Plant & fungus	1.01 (0.46, 2.20)	0.98 (0.94, 1.03)	2.08 (0.58, 7.46)	1.05 (0.99, 1.11)
Other/unknown	1.16 (0.44, 3.09)	1.04 (1.01, 1.07)	0.56 (0.24, 1.31)	0.97 (0.94, 1.00)

Abbreviations: RR, rate ratio; CI, confidence interval.
Note: Statistically significant RRs are indicated in bold.

© 2024 Noghrehchi F et al. *JAMA Network Open.*

eTable 6. Segmented Poisson Mixed-Effects Regression With AR(1)[a] to Investigate Changes in the Monthly Number of Self-Poisonings After the Implementation of Pesticide Bans

	**Change after 1st restrictions in level** RR (95% CI)	**Change after 1st restrictions in slope** RR (95% CI)	**Change after 2nd restrictions in level** RR (95% CI)	**Change after 2nd restrictions in slope** RR (95% CI)
**Proportional to agricultural chemicals**				
**Herbicide**	**0.84 (0.74, 0.96)**	1.00 (0.99, 1.00)	1.02 (0.87, 1.20)	0.99 (0.98, 1.00)
**Insecticide**	**1.10 (1.02, 1.19)**	1.00 (1.00, 1.00)	0.99 (0.90, 1.07)	1.00 (1.00, 1.01)
**Mixed/other/unknown**	0.99 (0.71, 1.40)	1.00 (0.99, 1.02)	1.07 (0.75, 1.51)	1.00 (0.98, 1.01)
**Proportional to all poisonings**				
**Herbicide**	**0.68 (0.55, 0.83)**	0.99 (0.98, 1.00)	1.07 (0.85, 1.35)	0.99 (0.98, 1.01)
**Insecticide**	**0.82 (0.71, 0.94)**	1.00 (0.99, 1.00)	0.95 (0.83, 1.09)	1.00 (1.00, 1.01)
**Mixed/other/unknown**	0.77 (0.55, 1.07)	1.00 (0.98, 1.01)	1.13 (0.80, 1.60)	1.00 (0.98, 1.01)
**Agricultural chemical**	**0.77 (0.68, 0.86)**	0.99 (0.99, 1.00)	0.99 (0.87, 1.11)	1.00 (0.99, 1.01)
**Household & industrial chemical**	1.16 (0.96, 1.41)	1.00 (0.99, 1.01)	0.96 (0.79, 1.17)	1.00 (0.99, 1.01)
**Medication**	**1.16 (1.05, 1.28)**	0.99 (0.98, 1.00)	0.98 (0.88, 1.08)	1.00 (1.00, 1.01)
**Plant & fungus**	1.17 (0.94, 1.46)	1.01 (1.00, 1.02)	0.91 (0.72, 1.15)	1.00 (0.98, 1.01)
**Other/unknown**	1.25 (0.89, 1.75)	1.00 (0.99, 1.01)	1.14 (0.82, 1.59)	0.98 (0.97, 1.00)

Abbreviations: RR, rate ratio; CI, confidence interval.
[a] AR(1): Autoregressive of order 1.
Note: Statistically significant RRs are indicated in bold.

© 2024 Noghrehchi F et al. *JAMA Network Open.*

eTable 7. Adjusted Segmented Poisson Mixed-Effects Regression With AR(1)[a] to Investigate Changes in the Monthly Number of Self-Poisonings After the Implementation of Pesticide Bans, Adjusted for Age and Sex

	**Change after 1st restrictions in level** RR (95% CI)	**Change after 1st restrictions in slope** RR (95% CI)	**Change after 2nd restrictions in level** RR (95% CI)	**Change after 2nd restrictions in slope** RR (95% CI)
**Agricultural chemical**	**0.81 (0.73, 0.9)**	0.99 (0.99, 1.00)	1.00 (0.90, 1.12)	1.00 (1.00, 1.01)
**Household & industrial chemical**	1.15 (0.94, 1.39)	1.00 (0.99, 1.01)	0.97 (0.80, 1.19)	0.99 (0.99, 1.01)
**Medication**	**1.15 (1.05, 1.26)**	0.99 (0.99, 1.00)	0.96 (0.88, 1.05)	1.00 (1.00, 1.01)
**Plant & fungus**	1.13 (0.91, 1.40)	1.01 (1.00, 1.02)	0.93 (0.74, 1.17)	1.00 (0.98, 1.00)
**Other/unknown**	1.26 (0.90, 1.78)	1.00 (0.99, 1.01)	1.13 (0.81, 1.58)	0.99 (0.97, 1.00)

Abbreviations: RR, rate ratio; CI, confidence interval.
[a] AR(1): Autoregressive of order 1.
Note: Statistically significant RRs are indicated in bold.

© 2024 Noghrehchi F et al. *JAMA Network Open.*

eTable 8. Segmented Poisson Mixed-Effects Regression With AR(1)[a] to Investigate Changes in the Monthly Case Fatality of Pesticide Groups After the Implementation of Bans

	**Change after 1st restrictions in level** RR (95% CI)	**Change after 1st restrictions in slope** RR (95% CI)	**Change after 2nd restrictions in level** RR (95% CI)	**Change after 2nd restrictions in slope** RR (95% CI)
**Agricultural chemical**	**0.32 (0.22, 0.46)**	1.00 (0.98, 1.02)	**1.78 (1.14, 2.76)**	1.01 (0.99, 1.03)
**Herbicide**	**0.40 (0.25, 0.62)**	0.99 (0.96, 1.01)	1.46 (0.47, 4.59)	1.02 (0.99, 1.06)
**Insecticide**	**0.31 (0.20, 0.50)**	1.01 (0.99, 1.03)	**2.01 (1.24, 3.26)**	0.99 (0.97, 1.02)
**Mixed/other/unknown**	0.54 (0.06, 5.22)	0.99 (0.91, 1.08)	2.53 (0.17, 38)[b]	0.95 (0.83, 1.1)

Abbreviations: RR, rate ratio; CI, confidence interval.
[a] AR(1): Autoregressive of order 1.
[b] Large CI due to low number of deaths in this category.
Note: Statistically significant RRs are indicated in bold.

© 2024 Noghrehchi F et al. *JAMA Network Open.*

eTable 9. Segmented Poisson Regression to Investigate Changes in the Monthly Number of Self-Poisonings After the Implementation of Pesticide Bans, Between January 2002 and December 2016

	**Change after 1st restrictions in level** RR[a] (95% CI)	**Change after 1st restrictions in slope** RR (95% CI)	**Change after 2nd restrictions in level** RR (95% CI)	**Change after 2nd restrictions in slope** RR (95% CI)
**Agricultural chemical**	**0.85 (0.78, 0.92)**	0.99 (0.99, 1.00)	0.99 (0.9, 1.1)	1.00 (1.00, 1.01)
**Household & industrial chemical**	**1.19 (1.04, 1.36)**	1.00 (1.00, 1.01)	0.9 (0.74, 1.09)	1.00 (0.99, 1.01)
**Medication**	**1.12 (1.02, 1.22)**	0.99 (0.99, 1.00)	0.99 (0.92, 1.07)	1.00 (1.00, 1.01)
**Plant & fungus**	0.87 (0.76, 1.00)	1.01 (1.00, 1.01)	1.09 (0.96, 1.24)	0.99 (0.98, 1.00)
**Other/unknown**	1.33 (0.96, 1.83)	1.00 (0.98, 1.01)	0.96 (0.71, 1.29)	0.99 (0.97, 1.00)

Abbreviations: RR, rate ratio; CI, confidence interval.
[a] RRs are adjusted for age and sex.
Note: Statistically significant RRs are indicated in bold.

© 2024 Noghrehchi F et al. *JAMA Network Open.*

eTable 10. Segmented Poisson Regression to Investigate Changes in the Monthly Case-Fatality of Pesticide Groups After the Implementation of Bans, Between January 2002 and December 2016

	**Change after 1st restrictions in level** RR (95% CI)	**Change after 1st restrictions in slope** RR (95% CI)	**Change after 2nd restrictions in level** RR (95% CI)	**Change after 2nd restrictions in slope** RR (95% CI)
**Agricultural chemical**	**0.33 (0.26, 0.42)**	1.00 (0.99, 1.01)	1.41 (0.99, 2)	**1.03 (1.02, 1.05)**
**Herbicide**	**0.39 (0.25, 0.6)**	0.99 (0.97, 1.01)	1.23 (0.61, 2.47)	1.02 (0.97, 1.07)
**Insecticide**	**0.31 (0.23, 0.4)**	1.01 (0.99, 1.02)	1.35 (0.9, 2.03)	**1.02 (1.01, 1.04)**
**Mixed/other/unknown**	1.02 (0.21, 5)	0.98 (0.92, 1.05)	1.1 (0.1, 11.8)[a]	1.07 (0.9, 1.27)

Abbreviations: RR, rate ratio; CI, confidence interval.
[a] Large CI due to low number of deaths in this category.
Note: Statistically significant RRs are indicated in bold.

© 2024 Noghrehchi F et al. *JAMA Network Open.*

eFigure 1. Flowchart of Patients in the Study

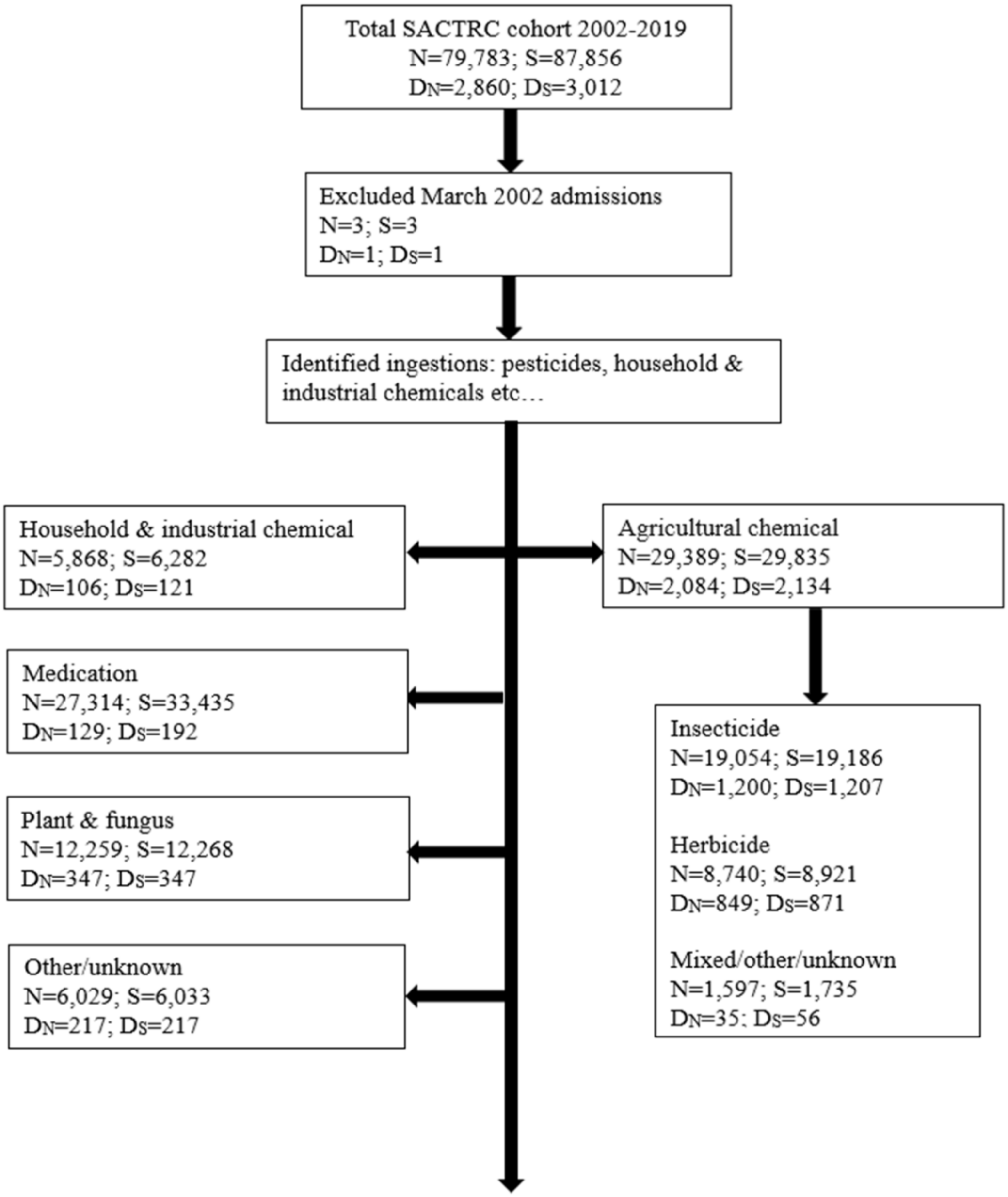

Abbreviations: N, Number of patients; S, Number of substances recorded (unknowns were recorded as one substance); D_N, Number of deaths; D_S, Number of deaths recorded against substances (deaths counted against each substance ingested).

© 2024 Noghrehchi F et al. *JAMA Network Open.*

eFigure 2. Timeline of Patient Recruitment Over Time

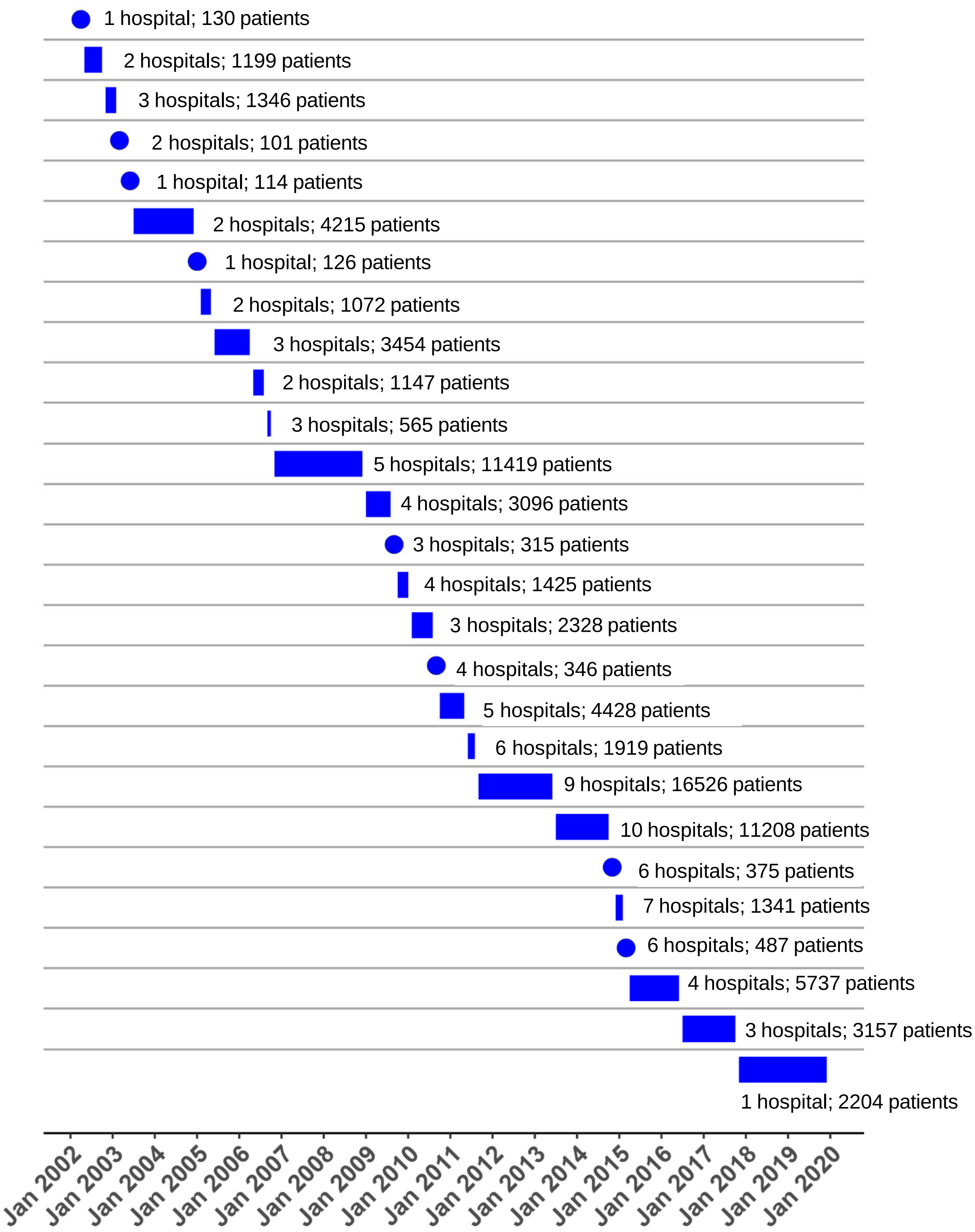

© 2024 Noghrehchi F et al. *JAMA Network Open.*

eFigure 3. Patient Recruitment Commenced on March 31, 2002, With Varying Periods Covered for the 10 Hospitals

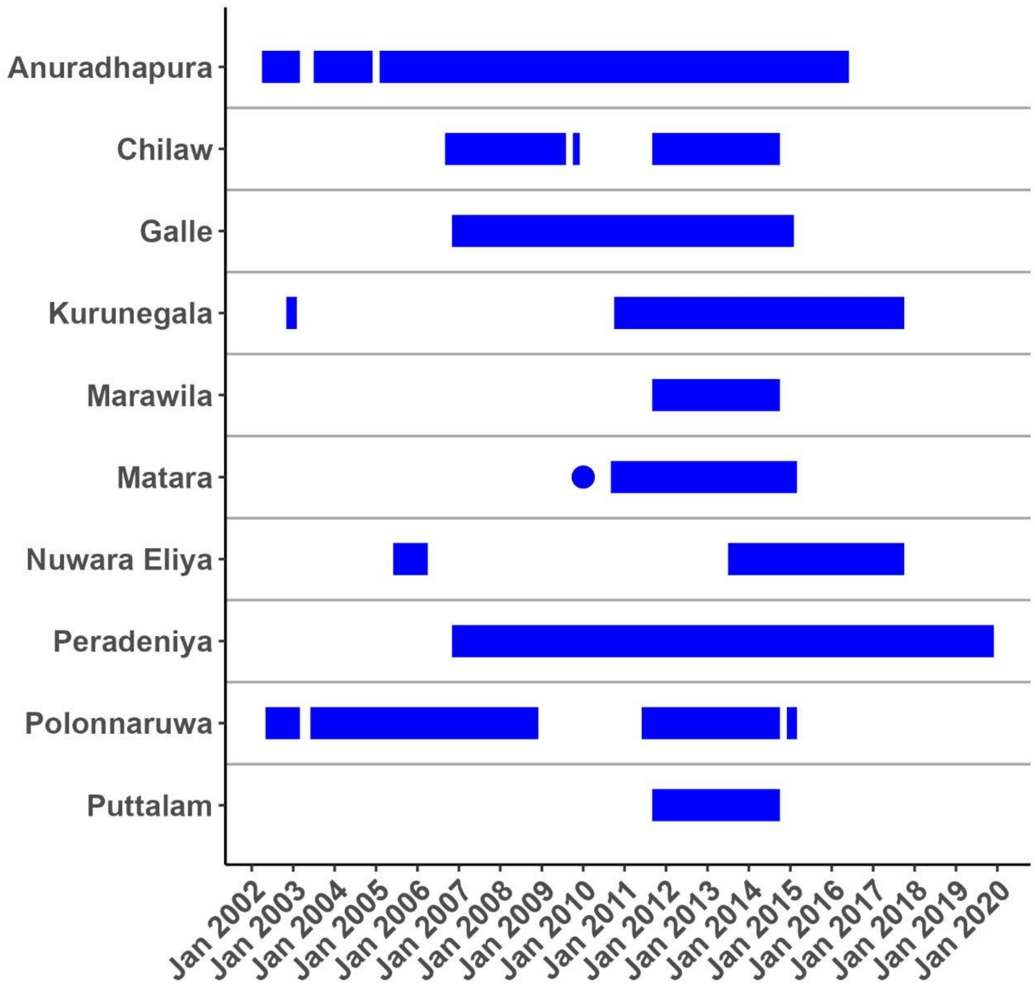

© 2024 Noghrehchi F et al. *JAMA Network Open.*

eFigure 4. Self-Poisoning Over Time by Different Hospital Sites

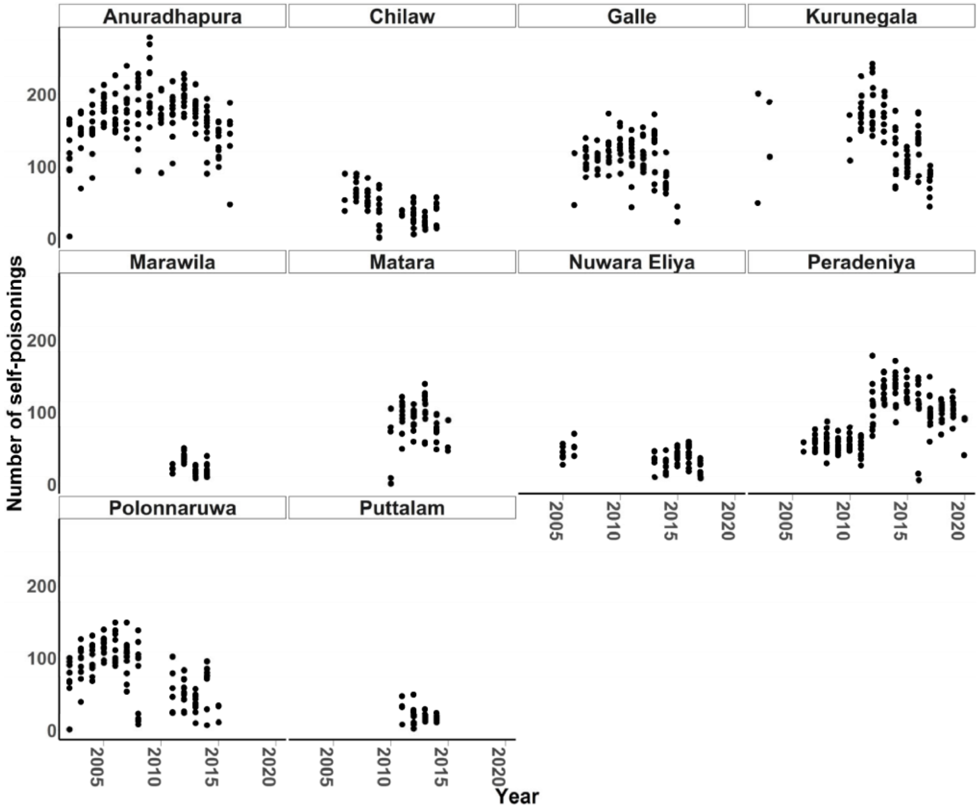

© 2024 Noghrehchi F et al. *JAMA Network Open.*

eFigure 5. Monthly Proportion of Self-Poisonings With Nonpesticides Before and After the Implementation of Pesticide Bans in Sri Lanka

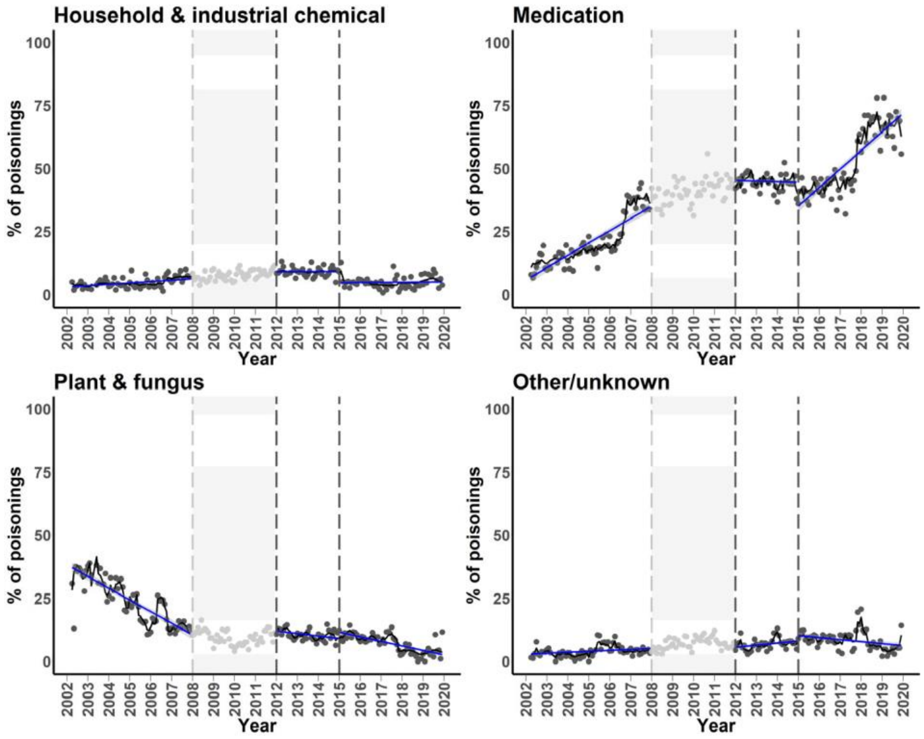

Note: Dashed lines indicate the time of restrictions. Shaded area between dashed lines indicates the start of first restrictions (washout period). Black lines are expected values from the segmented Poisson regression model. Blue straight lines are linear smoothing splines of the expected values.

© 2024 Noghrehchi F et al. *JAMA Network Open.*
