# Supplementary material for: Restrictions on Pesticides and Deliberate Self-Poisoning in Sri Lanka
Source: JAMA Netw Open. 2024 Aug 6;7(8):e2426209. doi: 10.1001/jamanetworkopen.2024.26209 (PMC11304112; doi:10.1001/jamanetworkopen.2024.26209)
Supplement: Supplement 2. — Data Sharing Statement [file jamanetwopen-e2426209-s002.pdf]

Data Sharing Statement

Noghrehchi. Restrictions on Pesticides and Deliberate Self-Poisoning in Sri Lanka. *JAMA Netw Open*. Published August 06, 2024. doi:10.1001/jamanetworkopen.2024.26209

Data
Data available: No

Additional Information
Explanation for why data not available: Individual patient data will not be made publicly available. We can provide the data dictionary for this study to anyone on request to the corresponding author. Individual de-identified participant data as described in this paper could be shared subject to governance and ethical approvals in Sri Lanka that ensure the data are only used in a manner consistent with the conditions and research purposes in the protocols under which the data was originally collected.
